# Staged Omental Wrapping for Perigraft Fluid Collection after Total Aortic Arch Replacement: A Case Report

**DOI:** 10.70352/scrj.cr.26-0473

**Published:** 2026-10-16

**Authors:** Gaku Tomita, Shinji Abe, Hikaru Tsuchida, Norihiro Ando, Yutaka Makino

**Affiliations:** Department of Cardiovascular Surgery, Oji General Hospital, Tomakomai, Hokkaido, Japan

**Keywords:** total aortic arch replacement, perigraft fluid collection, sterile inflammatory perigraft fluid collection, omental wrapping, staged surgical strategy

## Abstract

**INTRODUCTION:**

Perigraft fluid collection after aortic graft replacement is often self-limiting; however, progressive enlargement or the development of symptoms warrants careful evaluation. The differential diagnosis includes hematoma, abscess, seroma, and lymphatic leakage. However, differentiating among these conditions can be challenging based solely on clinical symptoms and imaging findings.

**CASE PRESENTATION:**

A 51-year-old man underwent total aortic arch replacement (TAR) for chronic Stanford type A aortic dissection. Nine months later, he developed rapidly progressive swelling and erythema over the upper portion of the median sternotomy incision. Contrast-enhanced CT revealed a low-attenuation fluid collection surrounding the prosthetic graft and extending into the subcutaneous mass. Although there was no evidence of active extravasation, anastomotic disruption, or gas formation suggestive of graft infection, exploratory resternotomy was performed under cardiopulmonary bypass (CPB) standby because a redo TAR might have become necessary if either anastomotic disruption or gross graft infection had been identified intraoperatively. Intraoperative inspection revealed no evidence of active bleeding. However, graft infection could not be definitively excluded because yellowish-brown turbid fluid was drained from the subcutaneous mass and around the prosthetic graft. Fluid samples were obtained for microbiological culture, and the perigraft space was thoroughly irrigated. Based on the intraoperative findings, fluid characteristics, and negative microbiological culture results, a sterile inflammatory perigraft fluid collection was considered the most likely diagnosis. Omental wrapping was subsequently performed as a second-stage procedure to manage the large residual space and prevent graft infection. No recurrence was observed during 12 months of follow-up.

**CONCLUSIONS:**

Perigraft fluid collection after aortic graft replacement has a broad differential diagnosis. When active bleeding or graft infection cannot be confidently excluded, an exploratory resternotomy under CPB standby may be a practical option for direct evaluation and management. Omental wrapping may be a simple and effective option for preventing recurrence and graft infection.

## Abbreviations

CPB
: cardiopulmonary bypass
FDG-PET
: ^18^F-fluorodeoxyglucose PET
LDH
: lactate dehydrogenase
TAR
: total aortic arch replacement
TG
: triglycerides

## INTRODUCTION

Perigraft fluid collection after aortic graft replacement is often self-limiting and rarely becomes clinically significant during follow-up. However, careful evaluation is required when the fluid collection enlarges or becomes symptomatic. Perigraft fluid collection has a broad differential diagnosis, including hematoma, abscess, seroma, and lymphatic leakage. Previous reports have demonstrated that these conditions are difficult to differentiate based solely on clinical and imaging findings.^1,2)^ Therefore, a safe and reliable diagnostic and management strategy is essential.

We report a case of perigraft fluid collection presenting as a subcutaneous mass after TAR. Exploratory resternotomy was performed under CPB standby, followed by staged omental wrapping. This strategy may be a practical option for safe evaluation and management when graft infection or active bleeding cannot be excluded preoperatively.

## CASE PRESENTATION

A 51-year-old man underwent TAR using a J Graft prosthesis and a Frozenix frozen elephant trunk (Japan Lifeline Co., Ltd., Tokyo, Japan) for chronic Stanford type A aortic dissection. Nine months after surgery, he presented with swelling and erythema at the upper portion of the median sternotomy incision.

The swelling had rapidly enlarged over several days. His medical history included chronic heart failure, hypertension, dyslipidemia, stage G3a chronic kidney disease (serum creatinine, 1.32 mg/dL; estimated glomerular filtration rate, 46.3 mL/min/1.73 m^2^), and type 2 diabetes mellitus, which was managed with oral semaglutide and dapagliflozin without the need for insulin therapy during hospitalization.

On admission, his body temperature was 36.5°C, blood pressure was 124/82 mmHg, and pulse rate was 81 beats/min. He was hemodynamically stable. Physical examination revealed an erythematous mass approximately 5 cm in diameter at the upper portion of the sternotomy incision (**Fig. 1**).

**Fig. 1:**
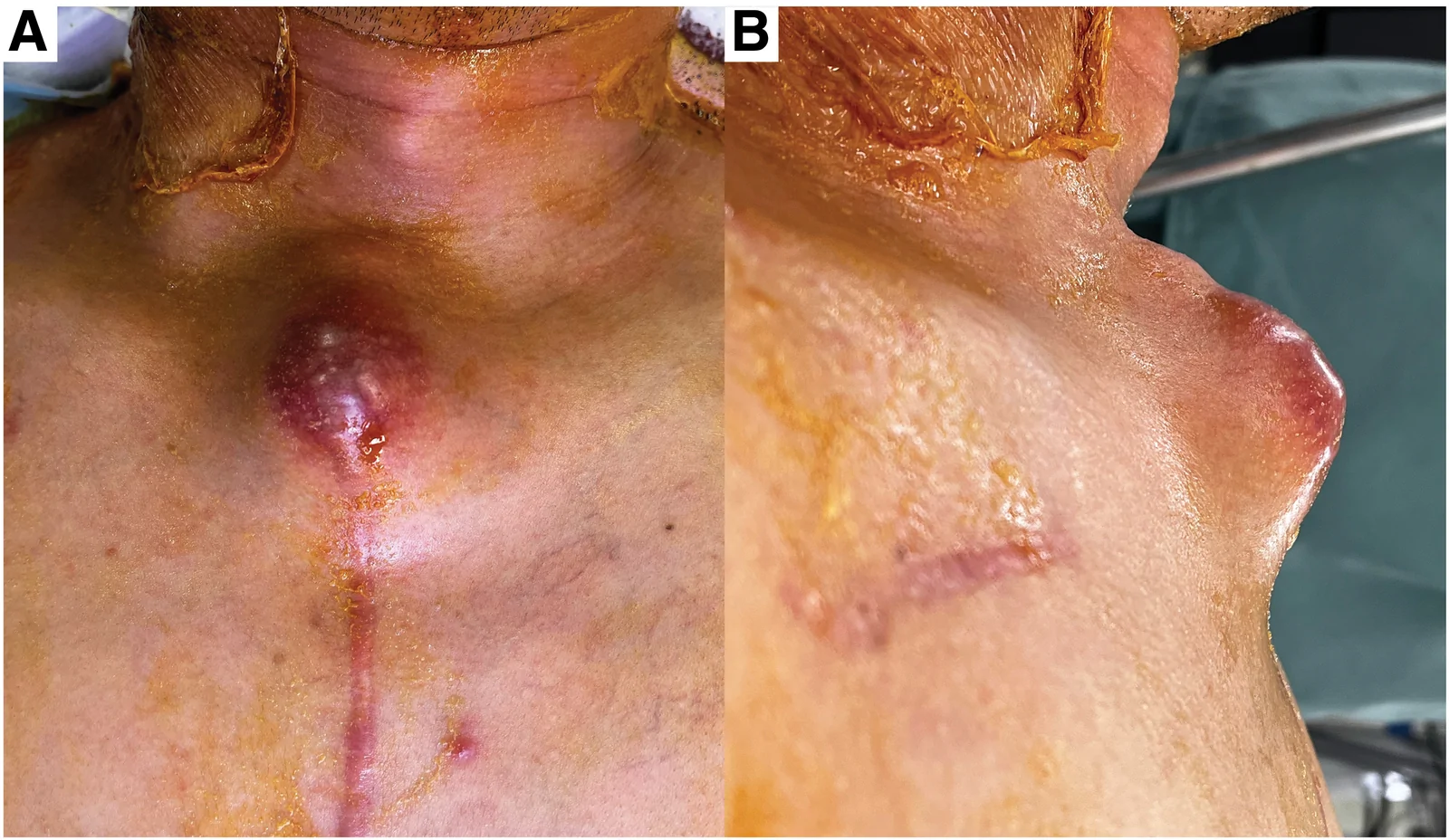
Subcutaneous mass at the upper median sternotomy site. (**A**) Anterior view showing a rapidly progressive erythematous subcutaneous mass at the upper portion of the sternotomy incision. (**B**) Lateral view showing an elevated subcutaneous mass with surrounding erythema.

Blood examination revealed a hemoglobin level of 16.4 g/dL, a white blood cell count of 6800/μL, a C-reactive protein level of 3.11 mg/dL, and a procalcitonin level of 0.07 ng/mL. After 2 sets of blood cultures were obtained, cefazolin (1 g every 8 h) was initiated empirically soon after admission.

Contrast-enhanced CT demonstrated a fluid collection surrounding the prosthetic graft and extending from the upper sternum to the subcutaneous mass. The mean attenuation of the perigraft fluid collection was 25 Hounsfield units, measured using polygonal regions of interest on 3 consecutive 3-mm axial slices, consisting of the slice showing the largest cross-sectional area and the adjacent cranial and caudal slices. No active extravasation or gas formation was observed (**Fig. 2**). Nevertheless, given the rapid enlargement of the mass, anastomotic disruption with active bleeding and graft infection could not be confidently excluded. Therefore, exploratory resternotomy was planned under CPB standby for both diagnosis and treatment.

**Fig. 2:**
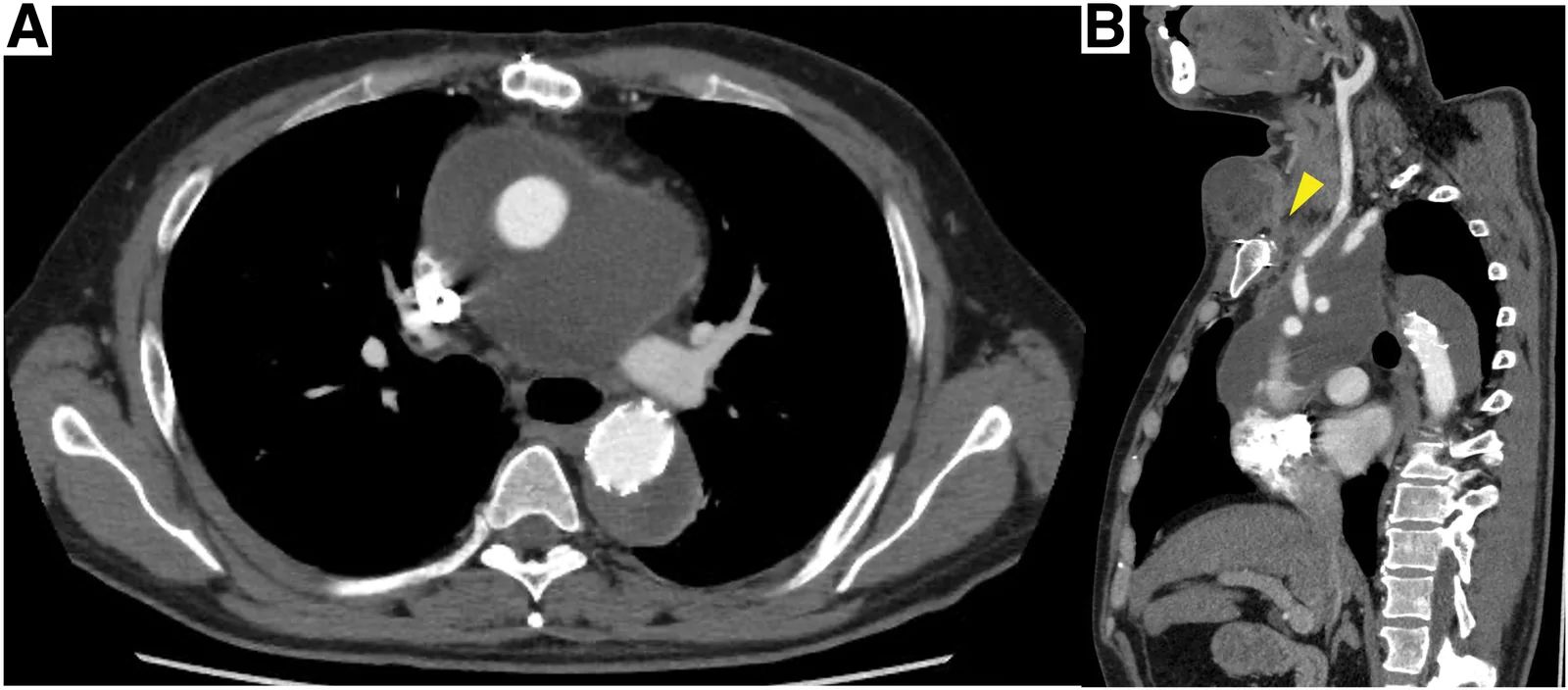
Preoperative contrast-enhanced CT. (**A**) Axial view showing a fluid collection surrounding the prosthetic graft. (**B**) Sagittal view showing communication between the perigraft fluid collection and the subcutaneous mass (arrowhead).

### Surgical management

#### First surgery: exploratory resternotomy and drainage

Resternotomy was performed on hospital day 2. The common femoral artery was exposed before resternotomy to allow rapid initiation of CPB in the event of critical bleeding. When the mass was opened, a yellowish-brown turbid fluid was drained (**Fig. 3**). After resternotomy, similar turbid fluid was found around the prosthetic graft. Careful inspection revealed no active bleeding at any of the anastomotic sites. In addition, no gross findings suggestive of deep sternal wound infection, including purulent collections around the sternal edges, necrotic granulation tissue, or sternal destruction, were observed. Fluid samples were collected for biochemical analysis and microbiological cultures. Because no active bleeding or gross graft infection was identified, redo graft replacement was not performed, and further treatment was deferred until the culture results became available.

**Fig. 3:**
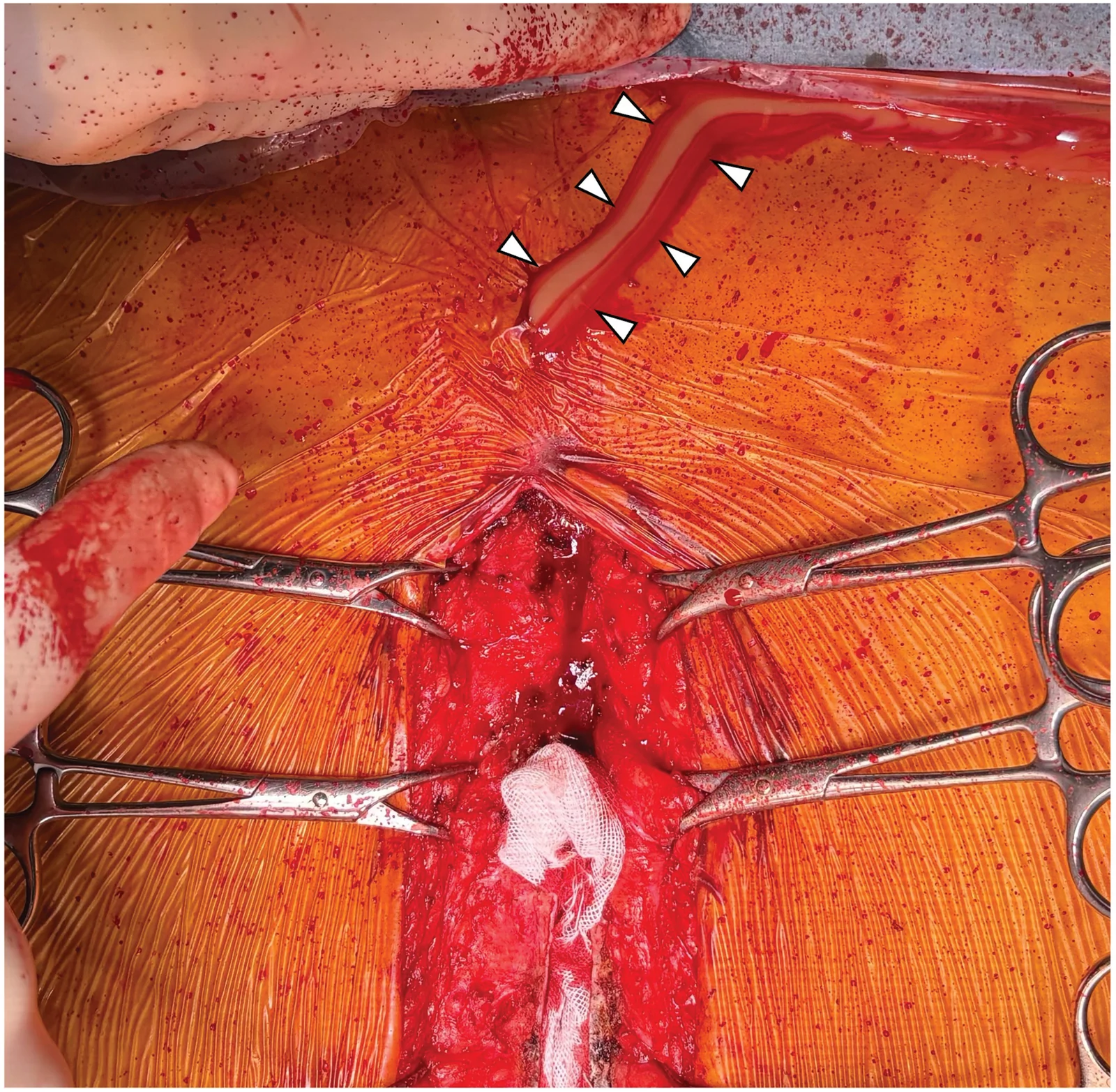
First surgery: Exploratory resternotomy. Turbid fluid draining from the subcutaneous mass (arrowheads).

Aerobic, anaerobic, and fungal cultures of the fluid specimen obtained intraoperatively were negative on POD 3 (hospital day 5), and blood cultures remained negative on POD 7 (hospital day 9). Fluid analysis showed semiquantitative leukocytes (3+), total protein 5.1 g/dL (serum, 7.3 g/dL), albumin 2.3 g/dL, LDH 14883 U/L (serum, 208 U/L), and TG 48 mg/dL. Glucose, total cell count, and differential cell count were not measured.

Based on these findings, anastomotic disruption with hematoma formation, which had been our primary concern, and graft infection were considered unlikely. The findings were considered most consistent with a sterile inflammatory perigraft fluid collection. Because the large residual dead space around the graft could lead to recurrence, omental wrapping was planned.

#### Second surgery: omental wrapping

After confirmation of the negative culture results, omental wrapping was performed 9 days after the initial operation (hospital day 11). Upon resternotomy, turbid fluid had reaccumulated around the prosthetic graft. A pedicled omental flap was harvested, transferred into the mediastinum through a small diaphragmatic window, and then wrapped around the prosthetic graft (**Fig. 4**). As no causative organism was identified, cefazolin was not continued as targeted antimicrobial therapy; rather, it was maintained as perioperative antimicrobial prophylaxis for surgical site infection for a total of 13 days, until POD 2 after the second operation (hospital day 13). CT performed on POD 7 confirmed circumferential coverage of the graft by the omentum. The patient was discharged home on POD 8. One month after the second surgery, CT revealed no fluid collection around the graft (**Fig. 5**). There was no recurrence during 12 months of follow-up.

**Fig. 4:**
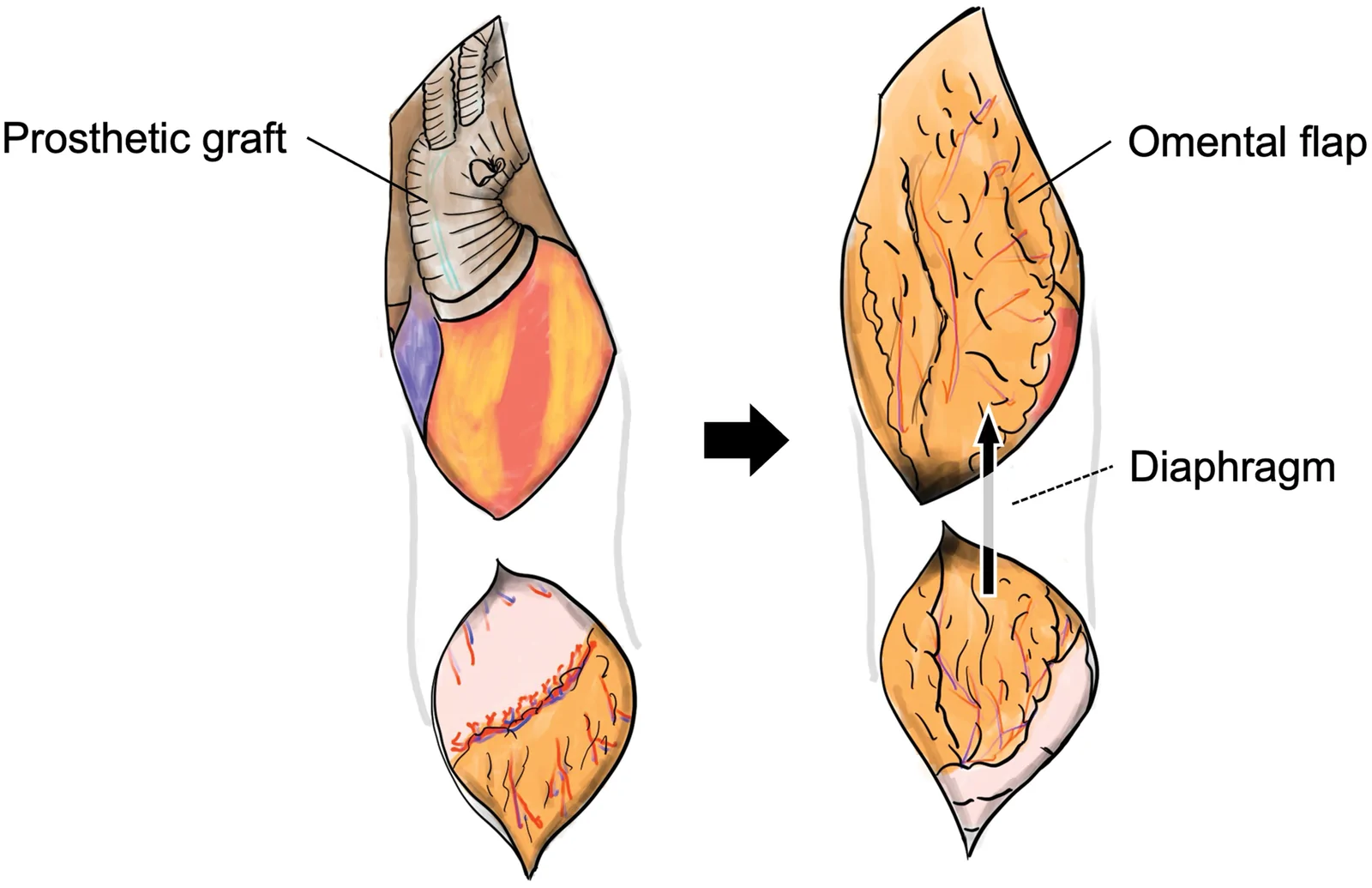
Second surgery: Omental wrapping. A pedicled omental flap wrapped around the prosthetic graft.

**Fig. 5:**
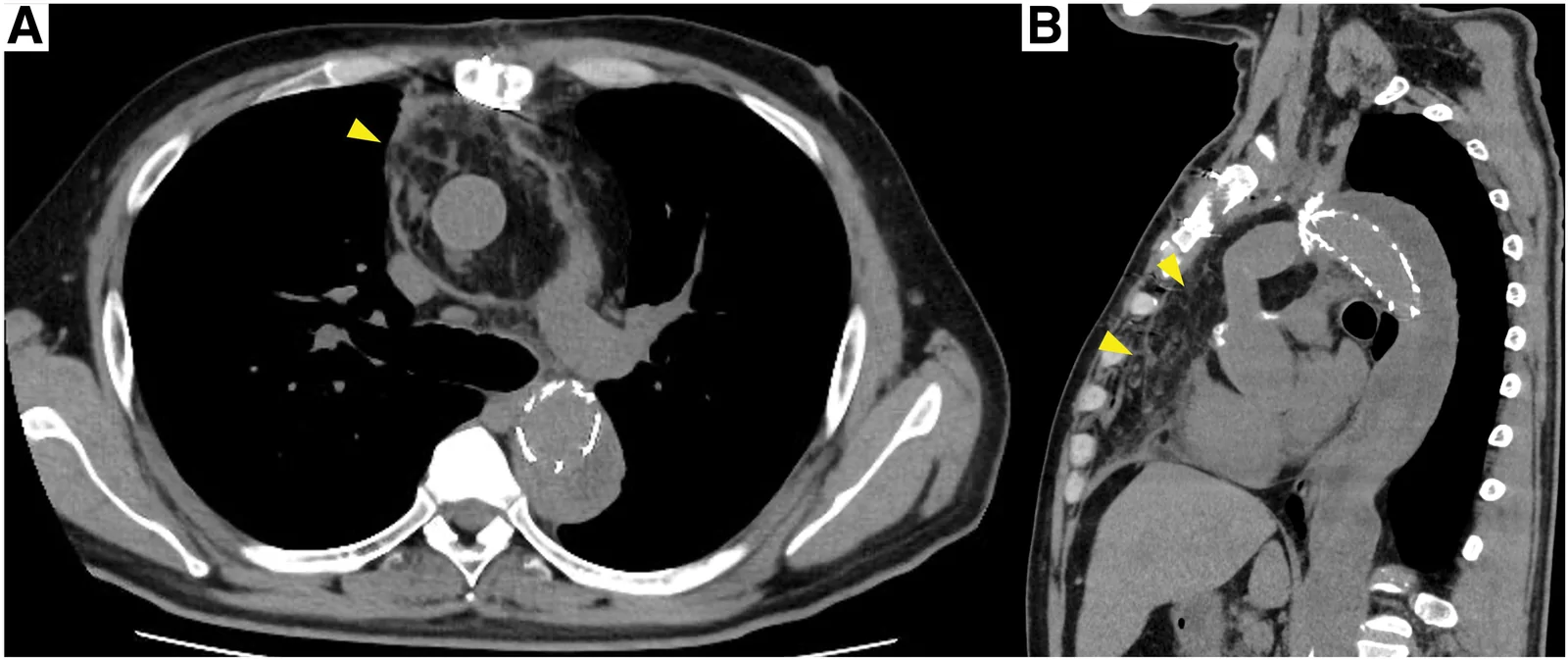
Postoperative CT at 1 month after surgery. (**A**) Axial view showing the omental flap (arrowhead) surrounding the prosthetic graft without evidence of recurrent fluid collection. (**B**) Sagittal view showing the omental flap (arrowheads) transferred anterior to the heart and wrapped around the prosthetic graft.

## DISCUSSION

The present case was characterized by a rapidly enlarging subcutaneous mass that was continuous with the perigraft fluid collection. Because active bleeding and graft infection could not be confidently excluded preoperatively, careful intraoperative evaluation was necessary.

### Differential diagnosis of perigraft fluid collection

Perigraft fluid collection after aortic graft replacement has a broad differential diagnosis, including abscess, hematoma, seroma, and lymphatic leakage.^1,2)^ Establishing a definitive preoperative diagnosis based solely on physical examination, imaging studies, and laboratory data is often challenging. Therefore, direct sampling of the fluid collection may be helpful when infection remains a diagnostic consideration.^2,3)^

The patient was afebrile preoperatively, had only mildly elevated inflammatory markers, and had no gas formation on CT suggestive of infection. Based on the intraoperative findings and negative culture results, obvious infection involving the superficial wound, sternum, or prosthetic graft was considered unlikely. However, low-grade infection caused by low-virulence organisms remained a possibility.

Hematoma was considered less likely in the absence of active extravasation on preoperative CT but remained a diagnostic consideration until intraoperative inspection during the first surgery confirmed the integrity of all anastomoses. Seromas typically contain serous fluid.^1)^ In contrast, the fluid in the present case was turbid and protein-rich, with a markedly elevated LDH level. These findings were not typical of a simple perigraft seroma. However, a perigraft seroma accompanied by inflammatory changes remained in the differential diagnosis.

The low TG level did not support chylous lymphatic leakage. However, lymphatic leakage could not be definitively excluded because lymphangiography was not performed.^4)^

Taken together, the present findings were considered most consistent with a sterile inflammatory perigraft fluid collection. Yamamoto et al.^5)^ reported 4 patients with a similar clinical presentation after thoracic aortic graft replacement. These patients developed subcutaneous masses 3–27 months after surgery and had culture-negative turbid perigraft fluid collections despite absent or only mild systemic inflammatory findings. The present case shared many of these clinical features, including delayed presentation with a subcutaneous mass, culture-negative, turbid perigraft fluid, and the absence of definitive systemic signs of infection. However, because histopathological examination and differential cell counts of the fluid were unavailable, and because fluid cultures were obtained after empirical cefazolin had been initiated, the diagnosis could not be established with certainty. Therefore, culture-negative infection and an inflammatory perigraft seroma remained in the differential diagnosis.

### Diagnostic approach to perigraft fluid collection

FDG-PET/CT is a useful adjunctive modality for evaluating vascular graft infection.^6)^ However, in the present case, FDG-PET/CT was not performed because surgical drainage was undertaken shortly after admission, allowing direct inspection of the perigraft space and microbiological sampling of the fluid.

In the present case, the rapidly enlarging erythematous mass raised concern for both active bleeding and infection involving the surgical wound or prosthetic graft. Because anastomotic disruption could not be confidently excluded, preoperative percutaneous aspiration was avoided to minimize the potential risk of major bleeding.

Instead, exploratory resternotomy under CPB standby was selected. This approach allowed direct inspection of the perigraft space and safe fluid sampling. Furthermore, if anastomotic disruption or gross graft infection had been identified intraoperatively, a redo TAR could have been performed immediately.

### Treatment of perigraft fluid collection

For perigraft fluid collections without graft infection or anastomotic disruption, several graft-preserving approaches have been reported, including drainage alone, fibrin glue application, endovascular relining, and omental wrapping.^7–10)^ In the present case, the fluid collection extended into the subcutaneous space, and a large residual dead space remained after drainage, suggesting that simple drainage alone would be insufficient to prevent recurrence. Endovascular relining was considered difficult because of the postoperative anatomy after TAR.^9,10)^

One-stage omental wrapping has been reported in selected patients for whom graft preservation was considered appropriate.^5,11)^ In the present case, anastomotic disruption was ruled out during the first operation. However, the turbid appearance of the perigraft fluid did not allow graft infection to be confidently excluded intraoperatively. Although complete removal of an infected prosthetic graft is required when graft infection is confirmed, such a procedure is highly invasive. Therefore, graft-preserving treatment was deferred until the culture results became available.

Negative-pressure wound therapy has been used as a bridge to a staged reconstruction in selected patients with graft infection.^12)^ However, in the present case, the mediastinum could be adequately irrigated and closed primarily, and no gross findings suggestive of mediastinitis or graft infection were identified intraoperatively. Therefore, we elected to perform primary chest closure with mediastinal drainage until the second surgery.

Once graft infection became less likely following the negative culture results, omental wrapping was performed to manage the residual dead space. Because the omentum has an abundant vascular and lymphatic supply and immunologic properties, it may help fill dead space and contribute to local control of inflammation or infection.^5,11,12)^ Therefore, omental wrapping was considered an appropriate graft-preserving treatment in this case.

## CONCLUSIONS

We report a case of perigraft fluid collection presenting as a subcutaneous mass after TAR. Perigraft fluid collection after aortic graft replacement can result from various etiologies, and assessment based solely on clinical and imaging findings may be difficult in some cases. In the present case, exploratory resternotomy was performed under CPB standby to allow direct inspection and fluid sampling because immediate redo TAR could have been required if anastomotic disruption or gross graft infection had been identified intraoperatively. Staged omental wrapping was subsequently performed to manage the residual dead space around the graft. This staged strategy may be a practical option for evaluating and managing perigraft fluid collections when graft infection or active bleeding cannot be confidently excluded preoperatively.

## DECLARATIONS

### Availability of data and materials

All data generated or analyzed during this study are included in this published article.
